# Personal restoration and feelings of guilt with victims of forced displacement in the colombian caribbean

**DOI:** 10.1192/j.eurpsy.2021.1917

**Published:** 2021-08-13

**Authors:** E.P. Ruiz Gonzalez, M.J. Arcos Guzman, M.N. Muñoz Argel, A. Uribe Urzola, J.D. Velez Carvajal

**Affiliations:** Psychology, Universidad Pontificia Bolivariana, Monteria, Colombia

**Keywords:** Personal restoration, feeling of guilt, forced displacement, victim

## Abstract

**Introduction:**

Forced displacement has been shown as a direct consequence of civil wars and armed confrontations, its effects on the victims are evidenced in the material, physical health and psychosocial effects (Mendoza, 2012; Pavas & Díaz, 2019; Ramos, 2018). It is common to identify in victims the presence of a post-offense emotional discomfort, which is recommended to work as a way of forgiveness for the achievement of personal restoration (Prieto & Echegoyen, 2015).

**Objectives:**

For this reason, the results of the study are presented, which has aimed to analyze the relationship between personal restoration and feelings of guilt with victims of forced displacement in the Colombian Caribbean.

**Methods:**

A correlational study has been carried out with a sample of 40 (n = 40) subjects of which 52.5% are men and 47.5% women, the mean age is 57.52 (σ = 13.591), all with a history of forced displacement; to the data collection has been used the CAPER instrument of Rosales, Rivera and Garcia (2017) (α = .592).

**Results:**

There is a positive bilateral correlation between the variables studied (r = .000; p = .829), the greater the personal restoration, the greater the sense of guilt is also manifested.

**Figure:**
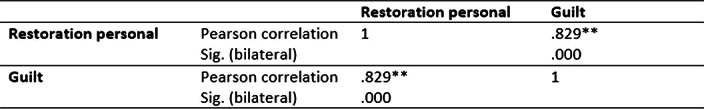

**Conclusions:**

For therapeutic work in personal restoration with victims of forced displacement, it is important to also include the feeling of guilt, which is presented as post-offense emotional distress.

**Disclosure:**

No significant relationships.

